# Supplementary material for: MetaRibo-Seq measures translation in microbiomes
Source: Nat Commun. 2020 Jun 29;11:3268. doi: 10.1038/s41467-020-17081-z (PMC7324362; doi:10.1038/s41467-020-17081-z)
Supplement: Supplementary file 10 — Supplementary Data 7 [file 41467_2020_17081_MOESM10_ESM.zip › File2/Confidence_High_Taxonomy/368151_out.krona.html]

Javascript must be enabled to view this page.

members
magnitude
magnitudeUnassigned
count
unassigned
taxon
rank

368151\_out

45

2
45
superkingdom

1239
45
phylum

186801
45
class

186802
order
45

family
2
186806

1730
2
genus

species
2

SRS062427\_contig\_number\_8064SRS1054716\_contig\_number\_13877
142586

186803
family
43


SRS012969\_contig\_number\_39701SRS013158\_contig\_number\_contig-100\_667.43173SRS014287\_contig\_number\_4436SRS015579\_contig\_number\_contig-100\_1017.155528SRS016989\_contig\_number\_15097SRS017103\_contig\_number\_38143SRS018656\_contig\_number\_10010SRS023526\_contig\_number\_39088SRS023715\_contig\_number\_23604SRS043667\_contig\_number\_4428SRS043768\_contig\_number\_contig-100\_744.192877SRS044535\_contig\_number\_25673SRS045244\_contig\_number\_5496SRS046068\_contig\_number\_1241SRS046717\_contig\_number\_8352SRS048164\_contig\_number\_13921SRS050998\_contig\_number\_15458SRS053335\_contig\_number\_17617SRS055017\_contig\_number\_36689SRS057478\_contig\_number\_5500SRS063190\_contig\_number\_10099SRS063370\_contig\_number\_contig-100\_47.87912SRS075773\_contig\_number\_22638SRS077294\_contig\_number\_19523SRS097958\_contig\_number\_23467SRS098061\_contig\_number\_contig-100\_640.93881SRS1055067\_contig\_number\_16838SRS142599\_contig\_number\_contig-100\_818.188973SRS143372\_contig\_number\_10888SRS144362\_contig\_number\_36697SRS144537\_contig\_number\_18601SRS146764\_contig\_number\_33321SRS147766\_contig\_number\_14750SRS147919\_contig\_number\_12115SRS147977\_contig\_number\_5723SRS149075\_contig\_number\_19218SRS893259\_contig\_number\_1282SRS893279\_contig\_number\_contig-100\_538.102593SRS893288\_contig\_number\_4745SRS893342\_contig\_number\_3893SRS893369\_contig\_number\_contig-100\_175.81100
39491
species
41

841
genus
2

2049040

SRS1055076\_contig\_number\_8171
1
species


SRS148091\_contig\_number\_11461
360807
species
1
